# Reviewed article: Santos ACJ, Quintino ND, Silva JEFA, Nunes MCP, Pereira JA, Sabino EC, Mota AF, Haikal DS. Hospitalizations due to stroke and their relationship with Chagasdisease: a population-based analytical study using data from the Hospital Information System of the Brazilian National Health System, Minas Gerais, Brazil, 2022. Epidemiol Serv Saude. 2026:35;e20240901

**DOI:** 10.1590/S2237-96222026v35e20240901.a

**Published:** 2025-11-21

**Authors:** Cristina Wide Pissetti

**Affiliations:** 1 Universidade Federal da Paraíba, João Pessoa, PB, Brazil

## Peer review A

## Questionnaire

Does the manuscript contain new and significant information to justify publication? Yes

Does the Abstract (Summary) clearly and accurately describe the content of the article? Yes

Is the problem significant and concisely stated? Yes

Are the methods described comprehensively? Yes

Are the interpretations and conclusions justified by the results? Yes

Is adequate reference made to other work in the field? Yes

## Manuscript Structure

Length of article is: Adequate

Number of tables is: Too few

Number of figures is: Adequate

Please state any conflict(s) of interest that you have in relation to the review of this paper. None

Interest: Excellent

Quality: Excellent

Originality: Excellent

Overall: Excellent

Recommendation: Minor Revision

## Comments

Prezados Autores,

Trata-se de um trabalho muito bem escrito e de suma importância.

Nossas sugestões:

1. Substituir a palavra-chave Internação Hospitalar por Hospitalização para adequar ao DECS;

2. No resumo, em métodos utilizaram-se registros do... e não utilizou-se;

3. Na página 4, substituir estimação por para estimar;

4. Na página 4, em “tal índice, proposto elo”: faltou o p em pelo;

5. Na página 5, linha 3, estimaram-se e não estimou-se;

6. Na página 7, linha 47, o apesar foi escrito a pesar;

7. Retirar a tabela suplementar 2;

8. A relação entre o perfil socioeconômico das regiões e os registros hospitalares merece maior detalhamento. Isso poderia contextualizar melhor as diferenças regionais observadas.

